# Is there a risk of prion-like disease transmission by Alzheimer- or Parkinson-associated protein particles?

**DOI:** 10.1007/s00401-014-1324-9

**Published:** 2014-07-30

**Authors:** Michael Beekes, Achim Thomzig, Walter J. Schulz-Schaeffer, Reinhard Burger

**Affiliations:** 1 Robert Koch-Institut, Nordufer 20, 13353 Berlin, Germany; 2 University Medical Center Goettingen, Robert Koch-Str. 40, 37099 Goettingen, Germany

**Keywords:** Alzheimer’s disease, Parkinson’s disease, Prion, Prion protein, Seed, Amyloid-β (Aβ), Tau, α-Synuclein, Transmission, Risk

## Abstract

The misfolding and aggregation of endogenous proteins in the central nervous system is a neuropathological hallmark of Alzheimer’s disease (AD), Parkinson’s disease (PD), as well as prion diseases. A molecular mechanism referred to as “nucleation-dependent aggregation” is thought to underlie this neuropathological phenomenon. According to this concept, disease-associated protein particles act as nuclei, or seeds, that recruit cellular proteins and incorporate them, in a misfolded form, into their growing aggregate structure. Experimental studies have shown that the aggregation of the AD-associated proteins amyloid-β (Aβ) and tau, and of the PD-associated protein α-synuclein, can be stimulated in laboratory animal models by intracerebral (i.c.) injection of inocula containing aggregated species of the respective proteins. This has raised the question of whether AD or PD can be transmitted, like certain human prion diseases, between individuals by self-propagating protein particles potentially present on medical instruments or in blood or blood products. While the i.c. injection of inocula containing AD- or PD-associated protein aggregates was found to cause neuronal damage and clinical abnormalities (e.g., motor impairments) in some animal models, none of the studies published so far provided evidence for a transmission of severe or even fatal disease. In addition, available epidemiological data do not indicate a transmissibility of AD or PD between humans. The findings published so far on the effects of experimentally transmitted AD- or PD-associated protein seeds do not suggest specific precautionary measures in the context of hemotherapy, but call for vigilance in transfusion medicine and other medical areas.

## Introduction

Many neurodegenerative diseases such as Alzheimer’s disease (AD), Parkinson’s disease (PD), Huntington’s disease, amyotrophic lateral sclerosis, or frontotemporal dementias share as a defining neuropathological feature the aggregation and deposition of misfolded endogenous proteins in the central nervous system (CNS). Although their amino acid sequences and native folds differ from each other, these disease-associated proteins form extracellular amyloid deposits or intracellular amyloid-like inclusions with common structural characteristics. Amyloid or amyloid-like protein deposits typically show a fibrillar morphology and an ordered “cross-β” assembly of the aggregated proteins. Comprehensive evidence suggests that a general mechanism of “nucleated growth” underlies the aggregation of endogenous proteins in the neurodegenerative diseases mentioned above (for a review see [16]).

A similar mechanism is thought to govern the self-propagation and cerebral deposition of amyloid-like beta-sheet-rich aggregates of misfolded prion protein (PrP) in transmissible spongiform encephalopathies (TSEs) [20, 87]. Yet, these fatal neurodegenerative diseases in animals [e.g., scrapie, bovine spongiform encephalopathy (BSE)] and humans [e.g., Creutzfeldt–Jakob disease (CJD)] were considered for many years unique. This is because they are caused, and can be transmitted between individuals, by unconventional pathogens devoid of coding nucleic acids designated as “proteinaceous infectious particles”, or “prions” [71, 72]. However, during the past few years an increasing number of prion-like phenomena have been detected in the context of AD, PD, and other non-prion neurodegenerative diseases [12, 32, 43, 52]. This has raised concerns that the transmission of pathological protein aggregates from common neurodegenerative diseases (which are generally presumed to be non-transmissible) may possibly pose a previously unrecognized risk to patient safety, e.g., in surgery or transfusion medicine.

To address this topical question for the two most frequent neurodegenerative disorders, AD and PD, this article reviews similarities and differences shown by pathological protein particles of these diseases in comparison to prions. After a brief introduction to prions and prion diseases, we provide an overview of animal experiments and epidemiological studies that have examined the transmission of protein aggregation, neurodegeneration or clinical disease between individuals by AD- or PD-associated protein particles. Finally, we discuss the results from these studies with respect to both implications for transmission risks and practical conclusions for transfusion medicine and other medical areas.

## The example of prions and prion diseases

### Molecular basis of prion formation, replication and transmission

Human TSEs, or prion diseases, occur in idiopathic, hereditary and acquired forms as exemplified by sporadic, familial and variant Creutzfeldt–Jakob disease (sCJD, fCJD and vCJD), respectively [73]. According to current knowledge, all of these diseases are caused and are transmissible by proteinaceous infectious particles (prions) that accumulate in the brain [71, 72, 87]. Prions are thought to be essentially composed of misfolded and aggregated conformational isomers of the cellular prion protein (PrP^C^), referred to as PrP^Sc^ or PrP^TSE^ [10, 72] (the acronym PrP^TSE^ will be used in this article). While PrP^C^ contains a high proportion of α-helical structure and only 3 % β-sheets, PrP^TSE^ has a profoundly different, amyloid-like conformation that is substantially made up of β-sheets and exhibits a markedly reduced amount of α-helical structure [15, 29, 66].

**Fig. 1:**
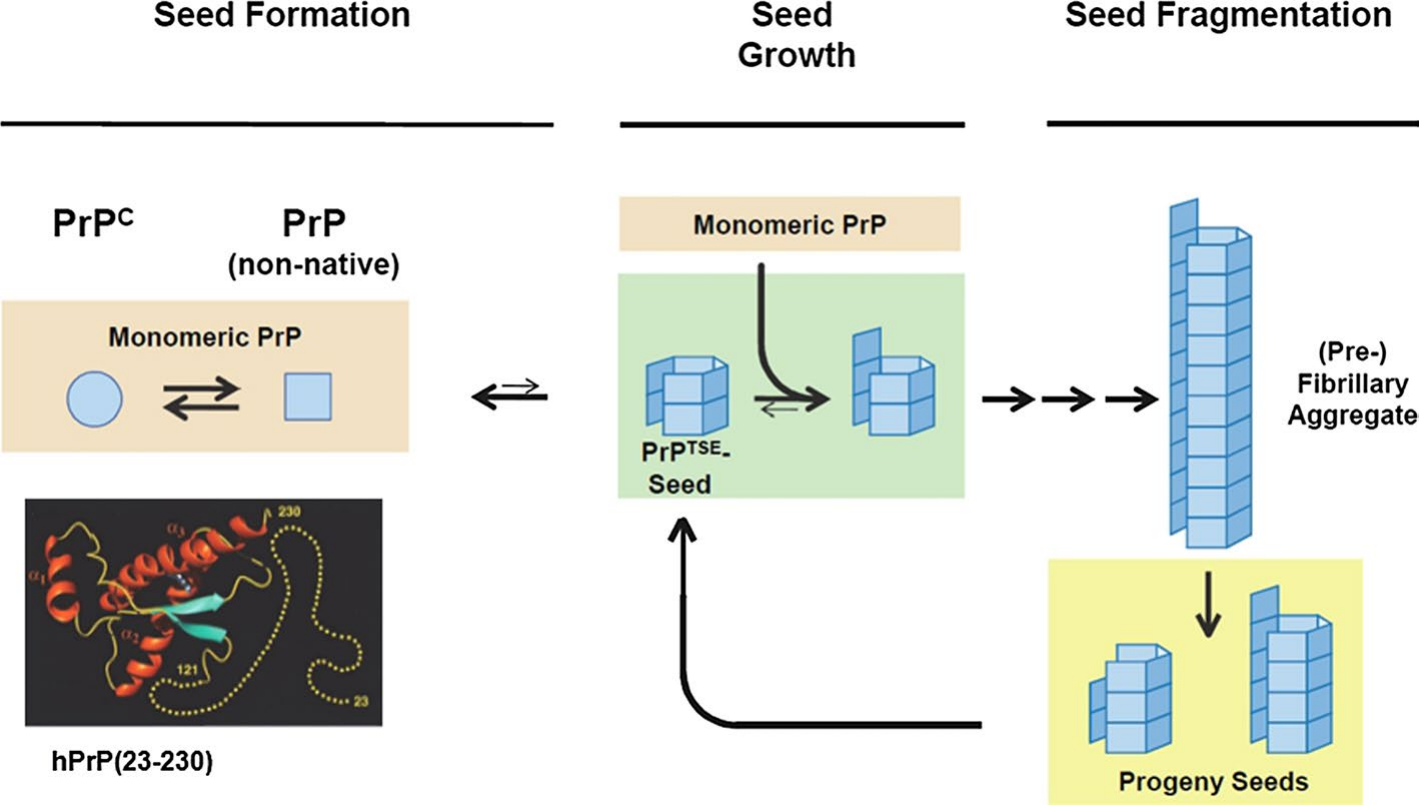
Mechanistic model of prion formation and replication by nucleation-dependent aggregation of the prion protein. The prion protein (PrP) can adopt different spatial structures. Cellular prion protein (PrP^C^) has a relatively high content of α-helical structure elements but may convert into isoforms with an aberrant conformation. Under certain conditions those PrP conformers can assemble into β-sheet-rich aggregates that constitute self-replicative proteinaceous infectious particles (prions). The misfolded and aggregated PrP in prions is referred to as PrP^TSE^. Primary seed formation, or nucleation, of PrP underlying the initial formation of prions in sporadic or hereditary prion diseases is controlled by a high kinetic barrier. However, once prions have been endogenously formed, or exogenously introduced into the body by infection, they behave as seeds that swiftly recruit and attach further PrP molecules. When prions eventually fragment into smaller PrP aggregates, progeny seeds enter the prion replication cycle and further propagate the pathological protein state. The NMR solution structure of recombinant human prion protein hPrP(23–230) was reproduced, in accordance with the copyright policies of PNAS, from Zahn et al. [97] (copyright by PNAS). Nucleation-dependent protein aggregation is thought to similarly underlie the formation and propagation of AD- and PD-associated protein particles

The prevailing mechanistic model of prion replication involves nucleation-dependent aggregation of the prion protein (Fig. 1) [20, 35, 87]. According to this concept, prions of sporadic or familial CJD are thought to be initially formed by a self-assembly of PrP monomers that occurs spontaneously (sCJD) or in association with stochastic (sCJD) or inherited (fCJD) amino acid exchanges in the prion protein which facilitate a structural PrP conversion. The de novo aggregation, or nucleation, of PrP constitutes a high kinetic reaction barrier [20, 35]. Thus, nucleation is considered as a slow and rate-determining step in the genesis of sporadic and hereditary prion diseases. However, once PrP nuclei have been formed the conversion of PrP^C^ to PrP^TSE^ is no longer kinetically inhibited. Then, the growth of PrP aggregates proceeds swiftly by the successive association of further PrP molecules, in an aberrant folding structure that reproduces the conformation of the PrP^TSE^ template, with the nucleus. This mechanism of nucleated growth is strongly reminiscent of the acceleration of crystallization by “seeding” (i.e., the addition of preformed “seeds” to a salt or protein solution). Accordingly, “PrP seeding” provides a straightforward explanation for why prions are agents that can transmit the misfolding and aggregation of PrP between individuals: Upon introduction into a new host they simply act as exogenous proteinaceous seeds which bypass the slow nucleation step of PrP conversion. When PrP^TSE^ aggregates fragment into smaller units this increases the number of prion protein particles with seeding-active growth surfaces. Thus, while the de novo formation of prions depends on nucleation, prion replication is mediated by an alternating growth and fragmentation of PrP^TSE^ aggregates. In seeded PrP^C^ to PrP^TSE^ conversion, the conformation of the PrP^TSE^ template is typically reproduced with high fidelity. On this basis, phenotypically distinct prion agents that are made up of PrP with an identical amino acid and referred to as prion “types” or “strains” can exist and propagate in vivo in the form of different PrP conformers [14, 45, 94].

Based on their replication mechanism prions can transmit misfolding and aggregation of PrP (i) at the molecular level, (ii) within or at the surface of cells, (iii) from one cell to another, (iv) in and between tissues [e.g., along neuroanatomical pathways in the peripheral and central nervous system (PNS, CNS), or between the lymphoreticular system and the PNS], and (v) also between individuals. The formation and/or replication of prions trigger pathogenic processes that are still poorly understood and cause neuronal degeneration in the brain. This results in neurological or mental symptoms and eventually fatal disease in sporadic, hereditary and acquired TSEs. Prions are able to transmit TSEs between animals, from animal to man (or vice versa), and between humans. Thus, they are genuinely infectious agents.

Notably, prion replication by the described propagation of PrP^TSE^ seeds is in principle indefinitely self-perpetuating. This holds true in cell-free conversion assays [6, 13, 93], cell assays [21, 48] and bioassays using transgenic or wild-type animals [31, 47] as evidenced by sustained PrP^C^ to PrP^TSE^ conversion and/or replication of infectivity in multiple serial passages in which the original seeds were gradually diluted out.

### Iatrogenic transmission of human prions: occurrence and prevention

A total of more than 450 cases of iatrogenic CJD have occurred worldwide after application of cadaver-derived human growth hormone (c-hGH) or gonadotrophin, transplantation of dura mater or cornea, and neurosurgery or EEG recording using invasive medical devices [11]. In addition, iatrogenic transmission of vCJD by blood transfusion is presumed for three clinically diseased recipients [36, 53, 95] and for one asymptomatic recipient [68] of non-leucodepleted erythrocytes. Furthermore, asymptomatic vCJD was diagnosed in a patient with hemophilia who had received factor VIII concentrate prepared from plasma pools that included donations from a vCJD-infected donor [67].

The BSE epidemic and resulting emergence of vCJD in the United Kingdom, and the subsequent occurrence of these novel TSEs in other countries, have substantially increased awareness of the zoonotic and iatrogenic risk potential of proteinaceous infectious particles. Together with the lessons learned from iatrogenic CJD this prompted comprehensive measures aiming at the prevention of iatrogenic prion transmissions to humans. An important element of these measures are the specifically devised safety guidelines on the reprocessing of surgical, diagnostic or other medical devices and on the supply of blood or blood products with a view to CJD and vCJD [39].

## Prions and Alzheimer- or Parkinson-associated protein aggregates: commonalities and dissimilarities

### Pathological protein aggregates in Alzheimer’s and Parkinson’s disease

The neuropathological hallmarks of AD are extracellular senile plaques and intraneuronal neurofibrillary tangles (NFT) primarily composed of aggregated amyloid-β [Aβ, a peptide processed from the cellular amyloid precursor protein (APP)] and partially hyperphosphorylated tau protein, respectively [60]. In about 80 % of patients, AD is associated with cerebral amyloid angiopathy, i.e., deposits of Aβ in blood vessels of the brain [84]. Tau aggregates in the brain do not occur exclusively in AD, but also in other neurodegenerative diseases which are collectively referred to as tau aggregation diseases and include frontotemporal dementias [41].

Brains of patients with PD typically show cell loss in the substantia nigra (particularly in the ventral part of the pars compacta) and contain fibrillar protein inclusions in cell bodies and processes of neurons. These inclusions are designated as Lewy bodies and Lewy neurites, respectively, and essentially consist of aggregated α-synuclein, a proportion of which is phosphorylated [23]. Such Lewy pathology also occurs in “dementia with Lewy bodies” (DLB), and pathological deposits of misfolded and aggregated α-synuclein primarily in oligodendrocytes are pathognomonic of multiple system atrophy (MSA), another α-synuclein aggregation disease [16]. Recently, it has been shown that in DLB and PD most α-synuclein aggregates occur in the presynapse of nerve cells [50, 81] and are linked with a synaptic failure [83]. It is under debate, whether Lewy bodies are a compartmentalization of protein aggregates to protect the cell from harmful (synaptic) α-synuclein aggregates [65, 82].

The deposition of pathological protein aggregates in the nervous system of humans with AD and PD shows partly stereotypic temporal–spatial spreading patterns, particularly for tau and α-synuclein [12, 43, 52], which is reminiscent of the spreading characteristics of prions. Studies in animals with an experimental or natural prion infection have shown that the deposition of PrP^TSE^ in the nervous system proceeds in highly defined temporal–spatial patterns, apparently transsynaptically, along neuronal projections and circuits from initial foci to adjacent and distant target areas [4, 5, 90]. Accordingly, stereotypic spatiotemporal patterns of pathological protein deposition in AD and PD can be explained by the formation of initial protein seeds early in pathogenesis, and a subsequent prion-like spread of misfolding and aggregation along neuroanatomical pathways. Alternatively, protein aggregation may start and proceed, in a relatively uniform temporal–spatial pattern, independently at different sites in the brain of AD and PD patients, and thereby produce the observed stereotypic courses [52]. The former hypothesis, however, seems to be supported by an observed spread of α-synuclein aggregation from affected brain tissue into fetal neuronal transplants in PD patients [43], and by further findings from in vitro and in vivo studies described in the following sections.

### Seeded aggregation of Aβ, tau and α-synuclein in vitro

The aggregation mechanisms of Aβ, tau and α-synuclein have been extensively explored in comprehensive in vitro studies which involved natural and recombinant protein forms [16]. Results from these studies indicate that the aggregation of Aβ, tau and α-synuclein occurs by a mechanism of nucleated growth which basically resembles that of PrP aggregation. However, it is still unclear to what extent AD- or PD-associated protein aggregates are self-propagating in a prion-like manner, i.e., how efficiently they fragment into progeny seeds, and how efficiently such progeny seeds then replicate in further cycles of growth and fragmentation.

During the past few years, it has become possible to mimic an indefinite self-perpetuation of the seeded aggregation of PrP (which is accompanied by a replication of infectivity) in vitro using cell-free serial protein misfolding cyclic amplification (sPMCA) [6, 13, 79, 93]. This technique reproduces putative key steps of prion replication in an accelerated manner in the test tube, and demonstrated the auto-seeding activity of newly generated PrP aggregates in multiple serial passages (i.e., after the initial seeding material had been successively diluted out). A similar, virtually indefinite, self-perpetuation of protein aggregation in cell-free conversion assays has not been shown so far for Aβ and tau aggregates, but was recently reported for α-synuclein based on findings in PMCA and aggregation assays that used in vitro generated fibrillar α-synuclein as seeding material [38, 77].

In cell assays, AD- and PD-associated protein aggregates show similarities to prions in terms of (i) binding to or uptake by cells of Aβ [96], tau [28, 63] or α-synuclein aggregates [63, 91], (ii) seeding in or on cells of Aβ [96], tau [63, 80] or α-synuclein aggregates [54, 63, 91], and (iii) intercellular spread of tau [28] and α-synuclein deposition [34]. In contrast with prions, aggregated Aβ and α-synuclein species have not yet been reported to show sustained self-propagation in multiple serial cell culture passages. However, Sanders et al. [80] recently demonstrated that different species of aggregated tau templated themselves with high fidelity through serial passages of HEK cell lines that stably expressed the aggregation-competent repeat domain of this protein.

The fact that AD- and PD-associated protein particles share nucleation-dependent protein aggregation as the molecular mechanism of their origin and other properties with prions, but not the latter’s obvious infectiousness (i.e., communicability or even contagiousness), is reflected in the term “prionoid” that has been introduced to designate prion-like seeding-active forms of Aβ, tau or α-synuclein [1, 2]. A substantial body of data obtained during the past few years [12, 32, 43, 52] has raised the question as to what extent Aβ-, tau- or α-synuclein aggregates behave like prions—and whether they can possibly transmit disease.

### Experimental transmission of Aβ-, tau- or α-synuclein aggregates to laboratory animals

Fatal prion infections can be experimentally transmitted from affected individuals to laboratory animals. Subsequently, they can be further passaged, in principle indefinitely, in vivo. During the past few years, similarly designed animal experiments have been increasingly performed to examine whether protein aggregation, neurodegeneration or clinical disease can result from an in vivo transmission of sample materials containing Aβ-, tau- or α-synuclein aggregates. In the following text, such sample materials are designated as “Aβ, tau or α-synuclein inocula”, or as “Aβ”, “tau” or “α-synuclein” with subscripted indices to indicate the origin of the respective inoculum.

**Fig. 2:**
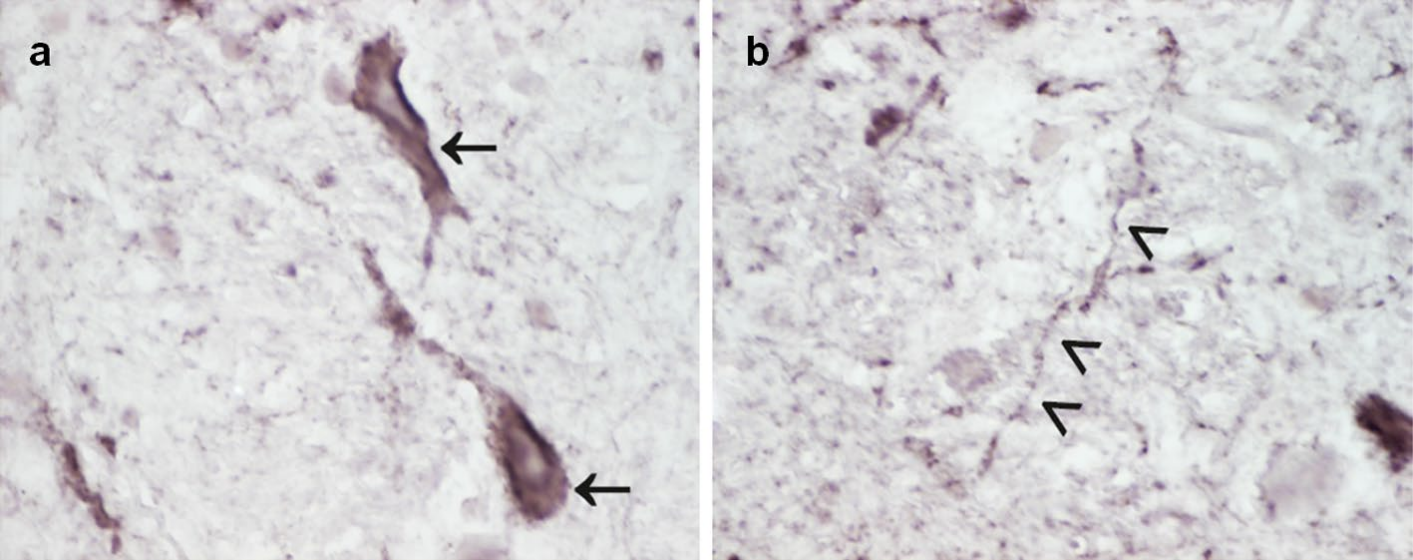
Congenital phenotype of cerebral α-synuclein aggregation in TgM83^+/+^ mice used for transmission studies with different α-synuclein inocula. TgM83^+/+^ mice expressing human A53T mutated α-synuclein begin to show α-synuclein aggregates in the CNS from an age of about 8 months. At the same age, few TgM83^+/+^ mice display signs of neurodegeneration and onset of eventually fatal motor dysfunction. The percentage of neuropathologically and clinically affected mice increases with age, and by 16 months of age all mice have developed the described phenotype [30]. The photomicrographs show pathological α-synuclein deposits in cerebral perikarya (**a**, *arrows*) and neurites (**b**, *arrowheads*) of a female TgM83^+/+^ mouse at an age of 257 days [immunostaining was performed using an anti-alpha-synuclein (phospho S129) antibody (ab51253; Abcam, Cambridge, UK)]. Using this mouse model, different studies were able to demonstrate that i.c. injected inocula containing α-synuclein aggregates from mice or humans accelerated the occurrence of intracellular α-synuclein inclusions in the CNS of the recipient animals [56, 62]. Additionally, these studies showed a premature onset of fatal disease in TgM83^+/+^ mice that had been challenged by i.c. injection of those murine inocula or fibrils that had been preformed in vitro from recombinant human α-synuclein
  that developed during their natural lifespan Aβ-, tau- or α-synuclein aggregates in the CNS, respectively. P301S PS19 and M83^+/+^ mice also developed neurodegeneration as well as severe motor dysfunction and fatal neurological disease.

For the experimental transmission of Aβ-, tau- or α-synuclein inocula, different types of wild-type animals were used, as well as transgenic rodents that homo-, hetero- or hemizygously expressed normal or mutated human APP-, tau- or α-synuclein. The mutated protein variants were associated with familial forms of AD, frontotemporal dementia, or PD. Many transgenic models used in these transmission studies showed a considerable overexpression of the respective proteins. A proportion (but not all) of the transgenic rodent models developed during their natural lifespan a congenital phenotype with (i) protein aggregation detectable in the CNS as Aβ deposits, inclusions of filamentous or NFT-like tau, or accumulations of α-synuclein, (ii) neurodegeneration in the CNS, and/or (iii) cognitive or neurological symptoms. Among the rodents challenged with Aβ-, tau- or α-synuclein inocula were the following animals (for study design, references and results see Table 1):

- Transgenic mice expressing mutated forms of human
  - APP (Tg2576 mice; APP23 mice; APP23:*Gfap*-luc mice; APP_Swe_/PSEN_ΔE9_ mice; APPPS1 mice),
  - tau (B6/P301L mice; P301S mice; P301S PS19 mice), or
  - α-synuclein (homozygous A53T M83^+/+^ mice, Fig. 2)

- Transgenic mice hemizygously expressing mutated human α-synuclein (A53T M83^+/−^: *Gfap*-luc mice) which were reported to show no protein aggregation or clinical disease until at least 629 days of age. It is, however, currently unclear whether these mice may develop protein aggregates, neurodegeneration or clinical disease at higher ages.
- Transgenic mice expressing normal forms of human APP (HuAPPwt mice) or tau (ALZ17 mice), and transgenic rats expressing a mutated form of human APP (APP21 rats). These transgenic rodent models did not show protein aggregates in the CNS during their (median) natural lifespan.
- Wild-type (wt) mice.

Two important aspects have to be considered for the interpretation of results from transmission studies in transgenic animals. First, if Aβ-, tau- or α-synuclein inocula stimulate the formation of cerebral protein aggregates in transgenic mice which have a congenital phenotype of protein aggregation, this leaves basically two alternative explanations: (i) that the inoculated material caused (i.e., transmitted) such aggregation in the true sense, or (ii) that the inoculum merely increased or accelerated the pathological protein deposition in specific brain regions in terms of a quantitatively stronger or earlier occurrence, respectively. Accordingly, it is deemed prudent to interpret a stimulation of protein aggregation in such bioassay animals conservatively as evidence for an “increase” or “acceleration” of the protein pathology [14].

Second, transgenic animals showing no congenital protein aggregation may theoretically still contain unnaturally high levels of different species of Aβ-, tau- or α-synuclein assemblies. Therefore, such mice “might be borderline with respect to the levels required for triggering higher-level aggregation. Increasing the Aβ (or tau- or α-synuclein—author’s note) load of a particular biochemical species could then trigger Aβ (or tau- or α-synuclein—author’s note) deposits” [70]. Accordingly, the occurrence of protein deposition in those transgenic animals after a challenge with Aβ-, tau- or α-synuclein inocula should be interpreted as a “triggering” of endogenous protein deposition.

Whether Aβ-, tau- or α-synuclein inocula can genuinely “cause” (i.e., actually transmit) protein aggregation in vivo, finally, requires testing in wild-type animals.

### Reported findings

Key findings from transmission experiments with Aβ-, tau- and α-synuclein inocula are described in the following paragraphs and summarized, for the studies that were performed in rodents, in Table 1.

**Table 1:** Effects of the transmission of sample materials containing Aβ-, tau- or α-synuclein aggregates to rodents

References	Study design			Effect on protein aggregation in the CNS				Spread of observed aggregation effect	Neurodegeneration in the CNS	Non-transient clinical abnormalities	Severe or fatal disease
	Inoculum	Route	Recipients	Increase	Acceleration	Triggering	Causation				
*Aβ*											
Kane et al. [46]	Aβ_hu-AD_	i.c.	Tg2576 mice		+			+	−^1a^	−^1a^	−^1a^
Meyer-Luehmann et al. [59]	Aβ_hu-AD_	i.c.	APP23 mice		+			+	n.d.	n.d.	n.d.
	Aβ_mu_	i.c.	APP23 mice		+			+	n.d.	n.d.	n.d.
	Aβ_mu_	i.c.	APPPS1 mice		+			+	n.d.	n.d.	n.d.
Bolmont et al. [8]	Aβ_mu_	i.c.	P301L mice^2a^		+			+	n.d.	n.d.	n.d.
Eisele et al. [26]	Aβ_mu_	i.c.	APP23 mice^3^		+			+	n.d.	n.d.	n.d.
Eisele et al. [27]	Aβ_mu_	i.p.	APP23 mice		+			+	n.d.	n.d.	n.d.
Rosen et al. [78]	Aβ_hu-AD_	i.c.	APP21 rats			+		–	n.d.	n.d.	n.d.
Morales et al. [61]	Aβ_hu-AD_	i.c.	HuAPPwt mice			+		+	n.d.	n.d.	n.d.
Stöhr et al. [88]	Aβ_mu_	i.c.	APP23:*Gfap*-luc mice	+				+	n.d.	n.d.	n.d.
	Aβ_preformed_	i.c.	APP23:*Gfap*-luc mice	+				+	n.d.	n.d.	n.d.
Duran-Aniotz et al. [25]	Aβ_hu-non AD_	i.c.	APPswe/PSENΔE9 mice	+				+	n.d.	n.d.	n.d.
Heilbronner et al. [37]	Aβ_mu_	i.c.	APP23 mice		+			+	n.d.	n.d.	n.d.
	Aβ_mu_	i.c.	APPPS1 mice		+			+	n.d.	n.d.	n.d.
*Tau*											
Clavaguera et al. [17]	tau_mu_	i.c.	ALZ17 mice			+		+	−^1b^	n.d.	n.d.
Clavaguera et al. [18]	tau_hu_	i.c.	ALZ17 mice			+		+	−^1c^	n.d.	n.d.
	tau_hu_	i.c.	C57BL/6 wt mice				+	+	n.d.	n.d.	n.d.
	tau_mu_	i.c.	ALZ17 mice			+		+	n.d.	n.d.	n.d.
Lasagna-Reeves et al. [51]	tau_hu_	i.c.	C57BL/6 wt mice				+	+	n.d.	−^1d^	−^1d^
Iba et al. [40]	tau_preformed_	i.c.	P301S PS19 mice		+			+	−^1e^	n.d.	−^1f^
Clavaguera et al. [19]	tau_mu_	i.p.	P301S mice	+				+	n.d.	n.d.	n.d.
Sanders et al. [80]	tau_mu_	i.c.	P301S PS19 mice		+			n. d.	n.d.	n.d.	n.d.
	tau_preformed_	i.c.	P301S PS19 mice		+			n. d.	n.d.	n.d.	n.d.
	tau_HEK cells_	i.c.	P301S PS19 mice		+			+^4^	n.d.	n.d.	n.d.
*α-Synuclein*											
Mougenot et al. [62]	α-syn_mu_	i.c.	TgM83^+/+^ mice		+			+	n.d.		+(Acceleration)
Luk et al. [55]	α-syn_preformed/mu_	i.c.	C57BL/6 wt mice				+	+	+(Causation)	+(Causation)	–^1j^
Luk et al. [56]	α-syn_mu_	i.c.	TgM83^+/+^ mice		+			+	+(Acceleration)		+(Acceleration)
	α-syn_preformed/hu_	i.c.	TgM83^+/+^ mice		+			+	+(Acceleration)		+(Acceleration)
Masuda-Suzukake et al. [58]	α-syn_hu-DLB_	i.c.	C57BL/6 wt mice				+	+	n.d.	n.d.	n.d.
	α-syn_preformed/mu_	i.c.	C57BL/6 wt mice				+	+	−^1g^	−^1h^	−^1h^
	α-syn_preformed/hu_	i.c.	C57BL/6 wt mice				+	+	−^1g^	−^1h^	−^1h^
Watts et al. [92]	α-syn_hu-MSA_	i.c.	TgM83^+/−^ mice		+			+	n.d.		+(Acceleration)
Guo et al. [33]	α-syn_preformed/hu_	i.c.	P301S P19 mice^2b^		+			+	n.d.	n.d.	n.d.
Recasens et al. [75]	α-syn_hu-PD_	i.c.	C57BL/6 wt mice				+	+	+(Causation)	+(Causation)	−^1i^

*Aβ-, tau- or α-syn* inocula containing Aβ-, tau- or α-synuclein aggregates, respectively (subscripted indices indicate the following origins: *HEK cells* HEK cell lines that stably expressed the aggregation-competent repeat domain of tau, *hu-AD* humans with AD, *hu-DLB* humans with DLB, *hu-non AD* humans without AD, *hu-MSA* humans with MSA, *hu-PD* humans with PD, *mu* mice, *preformed* in vitro preformed peptide or protein aggregates, *preformed/hu or preformed/mu* in vitro preformed aggregates of human or murine proteins, respectively), *n.d.* not determined, + positive finding reported, − negative finding reported, *i.c.* intracerebral administration to recipients, *i.p.* intraperitoneal administration to recipients

^1a−i^ Negative up to 5^a^, 15^b,c,g^, 11^d^, 6^e,h,j^, 9^f^ or 17^i^ months post-inoculation (g—negative finding refers to absence of dopaminergic degeneration)

^2a,b^ Indication of cross-seeding by Aβ_mu_ and α-syn_preformed/hu_ of mutated human tau, respectively

^3^ Intracerebral inoculation with Aβ_mu_ was performed by injection of liquid sample materials or implantation of steel wires

^4^ Positive finding with one of the tested HEK cell clones

### Aβ

When inocula containing Aβ aggregates from brain tissue of AD patients, non-demented elderly donors or transgenic mice with cerebral Aβ-amyloidosis (Aβ_hu-AD_, Aβ_hu-nonAD_, or Aβ_mu_, respectively), or inocula which contained Aβ aggregates that had been preformed in vitro from Aβ peptides (Aβ_preformed_) were

- intracerebrally injected into Tg2576- (Aβ_hu-AD_ [46]), APP23-(Aβ_hu-AD_ [59], Aβ_mu_ [26, 37, 59]), APP23:*Gfap*-luc-(Aβ_mu_, Aβ_preformed_ [88]), APP_Swe_/PSEN_ΔE9_- (Aβ_hu-nonAD_ [25]), APPPS1-(Aβ_mu_ [37, 59]), or B6/P301L mice (Aβ_mu_ [8]),
- intraperitoneally injected into APP23 mice (Aβ_mu_ [27]),
- or administered to APP23 mice by i.c. implantation of stainless steel wires that had been contaminated with Aβ_mu_ [26]
  this resulted in a stimulation of the cerebral Aβ deposition in the recipient animals which were all congenitally predisposed for protein aggregation. Specifically, an increase of the cerebral Aβ-burden was found in two mouse models (APP23:*Gfap*-luc mice injected with Aβ_mu_ or Aβ_preformed_ [88], and APP_Swe_/PSEN_ΔE9_ mice injected with Aβ_hu-nonAD_ [25]), while Tg2576 mice (injected with Aβ_hu-AD_ [46]), APP23 mice (injected with Aβ_hu-AD_ [59] or Aβ_mu_ [26, 37, 59]) and APPPS1 mice (injected with Aβ_mu_ [37, 59]) showed an acceleration of cerebral Aβ deposition.

this resulted in a stimulation of the cerebral Aβ deposition in the recipient animals which were all congenitally predisposed for protein aggregation. Specifically, an increase of the cerebral Aβ-burden was found in two mouse models (APP23:*Gfap*-luc mice injected with Aβ_mu_ or Aβ_preformed_ [88], and APP_Swe_/PSEN_ΔE9_ mice injected with Aβ_hu-nonAD_ [25]), while Tg2576 mice (injected with Aβ_hu-AD_ [46]), APP23 mice (injected with Aβ_hu-AD_ [59] or Aβ_mu_ [26, 37, 59]) and APPPS1 mice (injected with Aβ_mu_ [37, 59]) showed an acceleration of cerebral Aβ deposition.

Furthermore, the study in APP23:*Gfap*-luc mice demonstrated that preformed Aβ aggregates were sufficient to stimulate Aβ deposition in vivo [88]. In B6/P301L mice injected with Aβ_mu_ an acceleration of the formation of hyperphosphorylated intraneuronal tau inclusion was detected [8], which was indicative of cross-seeding. Also, intraperitoneally administered Aβ_mu_ was found to accelerate cerebral Aβ-amyloidosis [27], and increased or accelerated Aβ deposition in the brain was often detected beyond the site of inoculation in all studies. These latter finding resembled to some extent prion-like spreading phenomena. Finally, different morphotypes of Aβ aggregates were detected and differentially maintained upon inoculation into recipient animals [37]. Such morphotypes may provide yet another analogy to prions. However, the replication of distinct prion conformers can be maintained in vivo over multiple serial passages which has not been shown so far for Aβ.

Aliquots of Aβ_hu-AD_ were i.c. also injected into transgenic HuAPPwt mice [61] and APP21 rats [78] that had no congenital phenotype of protein aggregation. This triggered Aβ deposition, which would otherwise not have occurred, in brain tissue near the injection site, and, in HuAPPwt mice, also in distant brain areas.

However, none of the transmission studies with Aβ inocula provided evidence for cerebral neurodegeneration, pronounced cognitive decline or development of fatal disease in the challenged rodent models.

Primates appear as the most suited model animals for assessing potential hazards of Aβ inocula due to their close phylogenetic relationship to humans. Monkeys (marmosets) that had been i.c. inoculated with brain homogenates from nine patients with sporadic AD and five patients with familial AD were found in a long-term study (duration >20 years) to show more frequently and earlier cerebral Aβ deposition than control animals [3, 57, 76]. However, cerebral Aβ deposits also occurred in a proportion of control animals. In a conservative approach these findings would be interpreted as an indication that the injected inocula accelerated Aβ amyloidosis. Whether a triggering or causation of cerebral Aβ deposition had occurred in a proportion of the challenged primates could not be resolved by the experiment. In any case, none of the challenged monkeys showed the development of neurofibrillary tau deposits or AD typical signs of disease. The findings from this long-term study in nonhuman primates are consistent with the notion that cerebral Aβ deposits are an essential but not sufficient neuropathological criterion for AD, and with reports that the brain of elderly people who are not affected by neurodegenerative dementias may contain substantial deposits of aggregated Aβ [74, 85, 86, 89].

Similarly, the inoculation of sample material from more than 100 cases of AD into primates at the US National Institutes of Health (NIH) did not result in motor or behavioral abnormalities in the recipients that would have indicated a transmission of disease [9]. Additionally, neuropathological postmortem examinations of brain specimens from primates of the NIH study did not provide evidence for a pre- or subclinical disease transmission, according to a personal communication by members of the former NIH research team (P. Brown, D. Asher) reported by Irwin et al. [42]. In contrast to these negative findings with AD inocula, more than 300 cases of prion diseases were transmitted to primates at the NIH during the same period of time [9].

### Tau

The administration of inocula which had been prepared from brain tissue of transgenic mice with cerebral tau deposits (tau_mu_) or lysates of transgenic HEK cell lines that propagated tau inclusions (tau_HEK cells_), as well as the administration of inocula which contained tau aggregates that had been preformed in vitro from recombinant tau protein (tau_preformed_), by

- i.c. injection into P301S PS19 mice (tau_mu_ and tau_HEK cells_) [80]; tau_preformed_ [40, 80] or
- intraperitoneal injection into P301S mice (tau_mu_ [19])

accelerated or increased, respectively, the cerebral deposition of filamentous tau. The increase of tau aggregation in the brain of P301S mice which had been intraperitoneally injected with tau_mu_ indicated a centrifugal spread of tau aggregates or aggregation.

In addition, tau_mu_ [17, 18] or inocula containing aggregated tau from brain tissue of human patients with tau aggregation diseases (tau_hu_ [18]) were intracerebrally injected into transgenic ALZ17 mice. This triggered the initiation and spread of filamentous tau deposition in the brain of these recipient animals. The deposition of filamentous tau eventually extended from the injection sites to distant, anatomically connected brain regions.

I.c. injection of tau_hu_ into C57BL/6 wild-type mice caused inclusions of murine tau that were detected 6 months after inoculation at the injection site and subsequently increased over time [18]. When purified tau oligomers from AD brain tissue were intracerebrally injected into wild-type C57BL/6 mice, this induced widespread deposits of hyperphosphorylated murine tau filaments in the injected area and neighboring brain regions [51]. According to the authors of the latter study, AD-associated tau oligomers were thus shown to cause tau deposition in wild-type mice “via a mechanism reminiscent of that used by prions”.

Finally, serial transmission of tau seeding was observed in ALZ17 mice following the i.c. injection of homogenates prepared from ALZ17- or C57BL/6 brains that had been originally injected with tau_mu_ or tau_hu_, respectively [18]. Taken together, these findings suggested according to Clavaguera et al. [18] that “once tau aggregates have formed in discrete brain areas, they become self-propagating and spread in a prion-like manner”.

Most recently, Sanders et al. [80] reported that distinct species of tau aggregates could be stably propagated through multiple generations of transgenic P301S PS19 mice. These authors concluded that tau “encodes self-catalyzing conformational information that it propagates indefinitely with high fidelity”.

Despite the ability of tau inocula to increase, accelerate, trigger and cause tau aggregation in vivo, none of the transmission studies provided evidence for cerebral neurodegeneration, clinical abnormalities or severe disease in tau-challenged mice.

### α-Synuclein

When inocula containing aggregated α-synuclein from brain homogenates of aged TgM83^+/+^ mice that had developed cerebral protein aggregates and neurodegeneration as well as fatal clinical disease (α-synuclein_mu_) were i.c. injected into young asymptomatic TgM83^+/+^ mice, this accelerated the occurrence of intracellular α-synuclein inclusions in the brains of the recipient animals [56, 62]. The deposition of α-synuclein seemed to propagate through the CNS in a time-dependent manner along neural pathways and also occurred in the substantia nigra [56]. This suggested a parallel to both the “apparent spread of α-synuclein deposits in the human brain” [44], and the propagation of prions along neuroanatomical projections. After i.c. injection of α-synuclein_mu_, recipient animals eventually showed a reduction of tyrosine hydroxylase staining in nigral neurons with α-synuclein inclusions (suggesting impaired dopamine production in these cells) [56]. Additionally, TgM83^+/+^ mice that had been inoculated with α-synuclein_mu_ showed an accelerated development of severe motor dysfunction and premature death [56, 62]. After i.c. injection of inocula containing fibrils that had been preformed in vitro from recombinant human α-synuclein (α-synuclein_preformed/hu_), similar results were found in TgM83^+/+^ mice with respect to accelerated α-synuclein aggregation and premature onset of clinical disease [56].

Recently, Watts et al. [92] reported the experimental transmission of protein aggregation and fatal clinical disease for multiple system atrophy (MSA), an α-synuclein aggregation disease with α-synuclein inclusions in oligodendrocytes. In this study, inocula containing human α-synuclein aggregates from brain homogenates of MSA patients (α-synuclein_hu-MSA_) were intracerebrally injected into M83^+/−^: *Gfap*-luc mice [92]. Control M83^+/−^: *Gfap*-luc mice that had not been i.c. injected with α-synuclein_hu-MSA_ showed no protein aggregation or clinical disease (neurodegeneration was not examined) until at least 629 days of age. However, it is currently unclear whether such control mice may have developed a congenital phenotype of protein aggregation in the CNS and clinical disease at higher ages. Hemizygous TgM83^+/−^ mice that do not carry the *Gfap*-luc transgene develop pathological α-synuclein deposition in the CNS at 22–28 months of age. At the same age (660–840 days), many TgM83^+/−^ mice also develop the disease phenotype of homozygous TgM83^+/+^ mice [30]. Thus, a prudent approach would take the data reported by Watts et al. [92] either as an acceleration, or—if the absence of protein aggregation and clinical disease were shown to persist in M83^+/−^: *Gfap*-luc control mice during their natural lifespan—as a triggering of protein aggregation and clinical disease by the injected α-synuclein_hu-MSA_.

In another study, inocula containing two types of preformed fibrils produced in vitro from recombinant, C-terminally truncated human α-synuclein (α-synuclein_preformed/hu_) were intracerebrally injected into transgenic P301S PS19 mice [33]. While one type of the fibrils substantially accelerated cerebral tau deposition, possibly by cross-seeding, the other type of fibrils was much less able to do so. The two types of fibrils apparently represented distinct conformers of aggregated α-synuclein, and their different effects in terms of the stimulation of tau aggregation may be indicative of analogies to phenomena observed in the context of prion strains or prion types.

Furthermore, inocula containing fibrils that had been preformed in vitro from recombinant murine or human α-synuclein (α-synuclein_preformed/mu_ and α-synuclein_preformed/hu_, respectively), and inocula containing α-synuclein aggregates from enriched extracts of brain tissue from a patient with DLB (α-synuclein_hu-DLB_) or from patients with PD (α-synuclein_hu-PD_) caused cerebral deposition of endogenous α-synuclein in different types of C57BL6 wild-type mice after injection into the striatum (α-synuclein_preformed/mu_ [55]) or administration to the substantia nigra (α-synuclein_preformed/mu_, α-synuclein_preformed/hu_, α-synuclein_hu-DLB_ [58] and α-synuclein_hu-PD_ [75]). The subsequent spread of α-synuclein deposition seemed to follow neuronal connections and circuits. While the three studies in wild-type mice by Luk et al. [55], Masuda-Suzukake et al. [58] and Recasens et al. [75] produced similar results in these respects, they partly differed with regard to the occurrence of neurodegeneration and clinical abnormalities in the challenged animals.

Luk et al. [55] observed in their study a gradual loss of tyrosine hydroxylase immunoreactivity and neurons in the substantia nigra pars compacta, and reduced striatal dopamine levels in the injected hemisphere. This was accompanied by a performance deterioration in rotarod tests (latency to fall: ~200 s for α-synuclein_preformed/mu_-injected animals vs. ~325 s for control animals), and a poorer performance in wire-hang tests. However, gross motor or behavioral abnormalities were not observed, and overall motor activity was not significantly altered. In the study by Recasens et al. [75] the i.c. injection of α-synuclein_hu-PD_ caused a progressive nigrostriatal dopaminergic neurodegeneration. At 4 months post-inoculation these authors observed impaired motor ability when mice were examined in the pole test which measures motor coordination and balance. Yet, again, no gross motor or behavioral abnormalities were observed up to 17 months post-inoculation. Finally, Masuda-Suzukake et al. [58] reported findings suggesting that dopaminergic neurons were retained in the substantia nigra after injection of α-synuclein_preformed/hu_, while they observed a prominent reduction of the neurotransmitter enkephalin in some brain regions. In the study by Masuda-Suzukake et al. [58], no significant differences were observed in rotarod- or wire-hang tests between α-synuclein_preformed/hu_-injected and control mice at 6 months post-inoculation. At this time-point prominent cerebral α-syuclein deposition was already detectable in α-synuclein_preformed/hu_-injected mice.

Despite these partly divergent results, the studies by Luk et al. [55], Masuda-Suzukake et al. [58] and Recasens et al. [75] consistently failed to detect typical PD symptoms (such as tremor, rigor, slowness of movement or postural instability) in wild-type mice after the transmission of α-synuclein inocula.

Recasens et al. [58] also injected α-synuclein_hu-PD_ into the striatum or substantia nigra of rhesus monkeys. Striatal inoculation produced a widespread increase of α-synuclein immunolabeling and also an increase of phosphorylated α-synuclein species in the brain, while injection into the substantia nigra essentially resulted in diffuse local intracellular accumulations of α-synuclein. These α-synuclein pathologies were accompanied by a slowly progressing dopaminergic nigrostriatal neurodegeneration. At 14 months post-inoculation, when the study was terminated, the α-synuclein_hu-PD_-injected monkeys showed neither behavioral changes nor signs of clinical disease.

When samples from 24 cases of PD with dementia were inoculated into primates at the NIH, this produced exclusively negative results. The challenged primates did not show clinical signs of disease transmission [9]. Neuropathological postmortem examinations also failed to reveal indications for a pre- or subclinical disease transmission according to a personal communication by members of the former NIH research team (P. Brown, D. Asher) reported by Irwin et al. [42].

## Epidemiological studies on the transmissibility of Alzheimer’s and Parkinson’s disease

Available epidemiological data do not provide indications that transmission of AD or PD between humans occurs in real life. Observational and cohort studies failed to identify sufficient evidence for firm conclusions on the association of any modifiable risk factors for the development of AD [24, 69]. A limited number of studies specifically examined the epidemiological association between AD and blood transfusions. Findings of a study by Kokmen et al. [49] suggested that blood transfusions do not constitute a risk factor for AD in transfusion recipients. Two case–control studies by O´Meara et al. [64] and Bohnen et al. [7] which examined more than 550 cases and corresponding controls also did not find an association between blood transfusions and AD. This result was not influenced by the latency period between transfusion and occurrence of AD in transfusions recipients, the recipient’s apolipoprotein E epsilon 4 (APOE-e4) status, or the number of received transfusions. Finally, an epidemiological analysis in the United Kingdom of the causes of death of more than 6,000 hemophiliacs who had received blood products via plasma protein therapy throughout their whole life did not detect an elevated level of diseases of the nervous system in this group of patients [22].

A recent study by Irwin et al. [42] examined the risk of AD or PD transmission in recipients of cadaveric human growth hormone that had been prepared from the pituitary gland of deceased donors. Medical treatments with c-hGH caused more than 200 cases of iatrogenic Creutzfeldt–Jakob disease (iCJD) worldwide due to inadvertent contamination with infectious PrP^TSE^ seeds (i. e. prions) from donors who pre- or subclinically had incubated a prion disease [11]. Against this background, Iwrin et al. analyzed whether AD or PD occurred, similarly to iCJD, more frequently in recipients of c-hGH than in control persons. The authors reported that they found mild amounts of aggregated Aβ-, tau- and α-synuclein in the pituitary glands of normal individuals as well as of patients with neurodegenerative diseases. At the same time, the incidence of PD and AD exceeds that of human prion diseases in the general population (and thus probably also in c-hGH donors) by more than two or three orders of magnitude, respectively. Accordingly, recipients of c-hGH should have been exposed to potentially seeding-active Aβ-, tau- or α-synuclein particles with a much higher likelihood than to prions, provided that AD- or PD-associated protein particles passed like PrP^TSE^ seeds through the extraction procedure of c-hGH. Yet, Irwin et al. found in their epidemiological analysis that of 796 deceased c-hGH recipients none had developed AD or PD (mean age at death: 27.2 years [range 0–77 years]; mean duration from first treatment to death: 16.3 years [range 0–45 years]). Basically, this finding suggested two alternative conclusions: (i) That AD- or PD-associated protein particles were present in c-hGH but not able to transmit AD or PD to the hormone recipients, or (ii) that Aβ-, tau- or α-synuclein aggregates were more effectively removed or inactivated by the procedure for c-hGH purification than PrP^TSE^ seeds. In any case, no indications for a prion-like disease transmission by Aβ-, tau- or α-synuclein aggregates were detected in recipients of c-hGH, a group of patients that provide a “unique in vivo model of human-to-human transmission” [42].

## Discussion

### Transmission studies in animals

A substantial body of experimental data shows that the administration of inocula containing Aβ-, tau- or α-synuclein aggregates of animal, human or in vitro origin can increase, accelerate, trigger or cause the cerebral aggregation and deposition of endogenous Aβ-, tau- and α-synuclein in laboratory animals. As to the negative findings that have been published [9] or otherwise communicated [42] from the NIH’s primate study it should be noted that these experiments were performed before the modern tools to analyze neuropathological lesions of AD and PD were available. Thus, it appears tempting to speculate whether a re-examination of brain tissue specimens from these studies using current tools and antibodies (if possible at all) would, or would not, reveal the transmission of AD- or PD-like protein aggregation.

In contrast to the efficient stimulation of protein aggregation in vivo by Aβ-, tau- and α-synuclein inocula these sample materials have not been found to transmit severe or fatal diseases after i.c. injection into rodents and primates. Thus, in terms of disease transmission Aβ-, tau- and α-synuclein particles seem to fundamentally differ from prions. However, the failure to detect any transmission of a severe cognitive or neurological disease after i.c. injection of Aβ-, tau- and α-synuclein inocula may be due to the inability of the used animal models to reproduce cardinal symptoms of complex human neurodegenerative diseases such as AD or PD. Additionally, many transgenic mouse models were primarily engineered for the purpose of developing morphologically identifiable Aβ-, tau- or α-synuclein aggregates. Those mouse models often do not show significant CNS neurodegeneration (in proportion to the burden of protein aggregates), and whether they represent models of disease as opposed to protein aggregation is unclear. Although such transgenic models can be very sensitive to protein seeding by injected Aβ-, tau- or α-synuclein samples they may be rather insensitive to clinical disease transmission. In contrast to such transgenic mouse models of neuropathological AD or PD features, PrP transgenic mice were often specifically designed to increase the susceptibility to prion infections. This potential caveat has to be considered for AD or PD transmission studies in transgenic animals but does not apply to wild-type rodents or primates. Still, transmission studies in these latter animals also revealed a conspicuous discrepancy between efficient experimental acceleration (by Aβ inocula) or transmission (by tau- and α-synuclein inocula) of protein aggregation, and a lack of concomitant acceleration or transmission, respectively, of severe cognitive or neurological disease. Thus, the consistently failed disease transmissions by Aβ-, tau- and α-synuclein inocula observed so far may suggest an alternative conclusion: That Aβ-, tau- and α-synuclein aggregates, also when being seeding-active and partly able to produce neurotoxic effects, cannot transmit neurodegenerative diseases such as AD or PD.

In any case, the seeding effects that Aβ-, tau- or α-synuclein inocula have been shown to exert on endogenous proteins of laboratory animals have raised concerns of potential infectiological risks. A transfer of AD or PD protein particles between humans could hypothetically occur via contaminated and insufficiently reprocessed surgical instruments, or via blood or blood products.

### Epidemiological studies

Epidemiological studies can help to assess whether there are tangible risks for the transmission of AD or PD under real-life conditions. Available data from such studies do not provide indications for the transmissibility or transmission of AD or PD between humans via blood, blood products or other routes. However, AD and PD have a much higher prevalence than human prion diseases, and the interpretation of epidemiological studies on highly prevalent and multifactorial diseases such as AD and PD is complicated. Therefore, it may be difficult to epidemiologically track down any infectious origin if such cause underlay a sub-group of AD or PD cases.

### Conclusion and outlook

So far, neither experimental nor epidemiological studies provided evidence for a transmission of severe or even fatal disease by AD- or PD-associated protein particles. However, in the light of findings in TgM83 mice [56, 62, 92] it appears conceivable that exogenous α-synuclein seeds may accelerate the pathogenesis of genetically predisposed α-synuclein aggregation diseases. Furthermore, inocula that contained α-synuclein aggregates caused neurotoxic effects and neurological impairments in some studies with wild-type mice [55, 75]. Thus, a thorough decontamination of surgical instruments and other medical devices from aggregated Aβ-, tau- and α-synuclein by effective and routinely applicable reprocessing procedures may possibly add to patient safety. In contrast, the current data reviewed in this article do not suggest specific precautionary measures for hemotherapy.

Certainly, further epidemiological and experimental research is necessary to clarify the hazards possibly emanating from transmissible Aβ-, tau- or α-synuclein seeds in more depth. Yet, for the time being, prions of transmissible spongiform encephalopathies seem to remain unique pathogens.
